# Fanconi Anemia germline variants as susceptibility factors in aplastic anemia, MDS and AML

**DOI:** 10.18632/oncotarget.23328

**Published:** 2017-12-16

**Authors:** Bartlomiej Przychodzen, Hideki Makishima, Mikkael A. Sekeres, Suresh Kumar Balasubramanian, Swapna Thota, Bhumika J. Patel, Michael Clemente, Cassandra Hirsch, Brittney Dienes, Jaroslaw P. Maciejewski

**Affiliations:** ^1^ Department of Translational Hematology and Oncology Research, Lerner Research Institute, Cleveland Clinic, Cleveland, OH, USA

**Keywords:** Fanconi Anemia, MDS, AML, germline

## Abstract

Using next generation sequencing we have systematically analyzed a large cohort of 489 patients with bone marrow failure (BMF), including myelodysplastic syndrome (MDS), acute myeloid leukemia (AML), aplastic anemia (AA), and related conditions for the presence of germline (GL) alterations in Fanconi Anemia (FA) and telomerase genes. We have detected an increased frequency of heterozygous FA gene mutations in MDS and to lesser degree in AML suggesting that the presence of one normal allele may not be completely protective and indeed heterozygous FA lesions may have a long latency period before hematologic manifestation. In contrast, GL telomerase gene mutations were not associated with increased disease risk. When compared to large control cohorts, we have not detected an increased frequency of damaging variants among telomerase complex genes, including those previously believed to be involved in the pathogenesis of AA. Our results may suggest that while low penetrance and delayed disease onset can confound identification of genetic predisposition factors, GL FA alterations can be also associated with MDS.

## INTRODUCTION

Familial myelodysplastic syndrome (MDS) and aplastic anemia (AA) are rarely reported in adults and then usually in the context of early-onset diseases. In MDS for example, germline (GL) *RUNX1* mutations are associated with thrombocytopenia and subsequent clonal evolution [1, 2]. Similar findings have also been observed for GL *CEBPA* and *GATA2* [3–7]. A patient's family history may indicate inherited predisposition, but late onset, or incomplete penetrance often obscure assessment of hereditary factors and thus, aging or environmental exposures are implicated more often than genetic factors in the pathogenesis of sporadic MDS.

Fanconi Anemia (FA) is a prototypic inherited autosomal recessive bone marrow failure (BMF) syndrome with a highly variable penetrance [8]. Most of the mutations in FA gene pathway are found within three genes, *FANCA*, *FANCC* and *FANCG* [8]. FA cells exhibit hypersensitivity to DNA cross-linking agents, a laboratory marker of chromosomal instability [9] and FA patients frequently develop AA and MDS. While heterozygous carriers have been presumed to be asymptomatic in FA, various GL FA gene mutations have been suggested to increase susceptibility to breast cancer [10–13]. Dyskerin mutations are associated in autosomal dominant fashion with another hereditary BMF syndrome, dyskeratosis congenita (DC) [14], but mutations of other members of the telomerase complex, chiefly *TERT* and *TERC*, have been implicated in predisposition to both AA and MDS [15–18].

Based on the close association between AA and MDS as manifestations of congenital BMF syndromes, we hypothesized that both AA and heterozygous FA gene mutations as well as low penetrance telomerase gene alterations may represent risk factors for otherwise typical, sporadic adult MDS and AA. For that purpose we have systematically screened the open reading frames of FA and telomerase complex genes to identify and map both novel and established FA sequence alterations.

## RESULTS

### Identification of germline variants in Fanconi Anemia and telomerase complex genes

**Figure 1:**
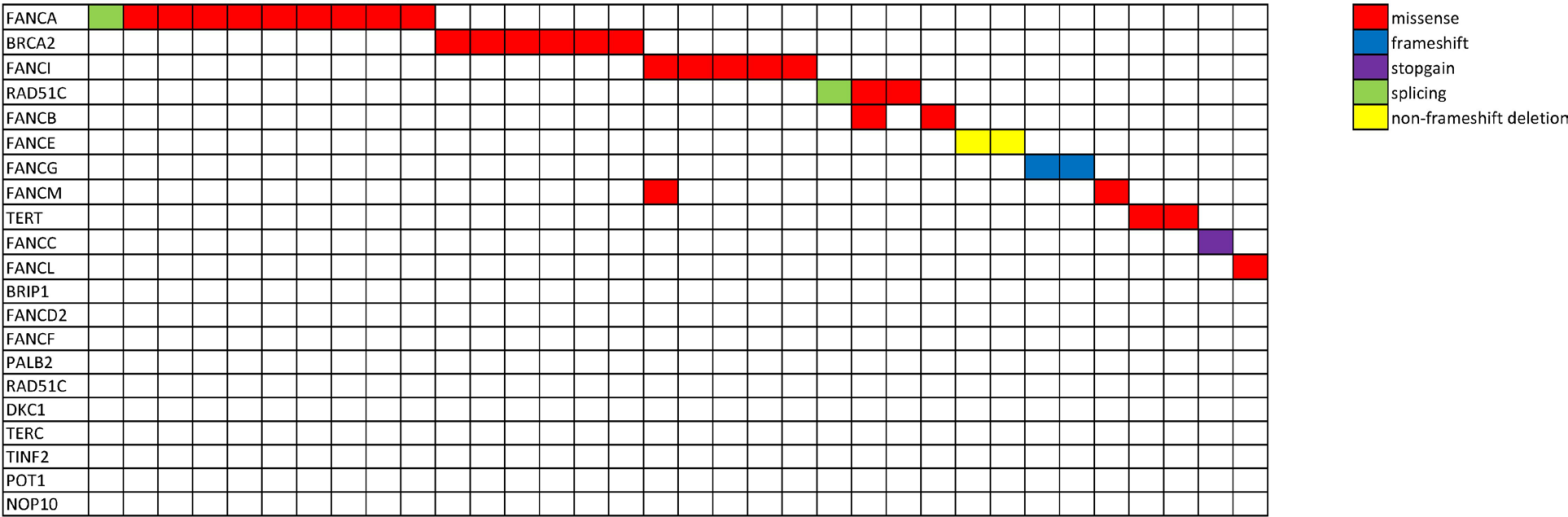
Mutational spectrum of patients harboring Fanconi Anemia and telomerase germline variants Schematic representation of the cohort of patients carrying germline variants in Fanconi Anemia and telomerase complex genes. Each row represents a gene and each column represents an individual patient (*N* = 34). Two patients harbored two variants each. Each color encodes a different type of mutation (red, missense; blue, frameshift; purple, stopgain; green, splicing variant; yellow, non-frameshift deletion).

All coding frames of 14 FA and 5 telomerase genes (Supplementary Table 1) were sequenced in a cohort of patients with BMF (*N* = 489), including MDS/sAML (*N* = 305), Myeloproliferative neoplasms (*N* = 33), and AA/paroxysmal nocturnal hemoglobinuria (PNH) (*N* = 151; Table 1). For the purpose of this analysis, the candidate alterations were sub-classified to prioritize subsequent analytic steps (Supplementary Figure 1); *tier-1* lesions included known disease-prone sequence alterations/mutations (OMIM [19]/Rockefeller Fanconi mutation database [20]), new nonsense or frameshift mutations, and exceedingly rare, individual, missense mutations (<0.01%, no reported homozygotes form in the control cohort), all of which are predicted as deleterious by >4/6 applied scoring algorithms (PolyPhen2 [21], PhyloP [22], SIFT [23], LRT [24], MutationAssesor and MutationTaster [25] within Annovar [26]). All *tier-1* alterations were screened to exclude somatic lesions and technical artifacts. AML Tier-1 variants’ germline status was confirmed using CD3 derived DNA, where specimen was available. *Tier-2* lesions were defined as variants with a high general population frequency of >0.01% and were not studied further. Instead, our subsequent analysis focused on *tier-1* lesions. In total, we found 36 *tier-1* gene mutations including 2 recurrent frameshift *FANCG* mutations. We also found 2 splicing variants (*RAD51C* and *FANCA*) and one stop gain variant (*FANCC*) (Figure 1, Supplementary Table 2). Only two variants among 36 reported were related to telomerase pathway and both were located on *TERT* gene. Among the genes tested, the most frequent SNVs were found in *FANCA* (*N* = 10) and *BRCA2* (*N* = 6). Overall, carriers of heterozygous *FANCA* alterations showed an increased risk of developing MDS (OR 4.9, *P* < 0.001), patients with *FANCG* showed an increased risk of developing MDS (OR 5.9, *P* < 0.05) and patients carrying *FANCE* variants tended to show a stronger risk for development of MDS although not statistically significant (OR 4.9, *P* < 0.10). RAD51C was the only gene which was nominally associated with development of AA (OR 6.9, *P* < 0.05). When averaged rates of total number of FA gene variants were compared to a healthy cohort of samples from ExAC database, we could find statistically significant differences for selected genes only, with varying frequencies among different disease subgroups. *FANCA*, *FANCE* and *FANCG* gene were present at higher rates among MDS subcohort (Table 2). Overall there was a higher rate of FA variants among disease subgroups, when compared with healthy population, but when looked closely it was driven only by a handful of genes within that pathway (Table 2).

**Table 1:** Clinical characteristics of patients participating in the study

Patient characteristics	% (*n*/range)
Median age (years, median, range)	65 (9–83)
Sex (male)	56% (254)
Type of disease	
MDS	48% (233)
MDS low risk	27% (130)
MDS high risk	14% (70)
MDS/MPN	7% (33)
sAML	15% (72)
AA+AA/PNH+PNH	31% (151)
total	489
Karyotype (MDS and AML only)	
Normal	33% (114)
del(5q),-5	10% (33)
del(7q),-7	10% (33)
-Y	2% (8)
del(20q),-20	6% (21)
trisomy 8	4% (14)
Complex (≥3)	17% (57)
Others	21% (73)
TOTAL	344

**Table 2:** Variant distribution among different patient population, compared with general, healthy population (ExAC)

Gene	AA	OR_AA	Pval_AA	MDS	OR_MDS	Pval_MDS	AML	OR_AML	Pval_AML	ExAC
*BRCA2*	1/151	0.72	0.59683	3/233	1.41	0.47748	2/72	3.09	0.14243	306/33370
*BRIP1*	0/151	N/A	1.00000	0/233	N/A	0.63722	0/72	N/A	1.00000	174/33370
*FANCA*	1/151	0.82	0.65464	9/233	**4.93**	**0.00016**	0/72	N/A	1.00000	270/33370
*FANCB*	2/151	2.45	0.20129	0/233	N/A	0.64111	0/72	N/A	1.00000	182/33370
*FANCC*	0/151	N/A	1.00000	1/233	2.21	0.36851	0/72	N/A	1.00000	65/33370
*FANCD2*	0/151	N/A	1.00000	0/233	N/A	0.63479	0/72	N/A	1.00000	168/33370
*FANCE*	0/151	N/A	1.00000	2/233	5.00	0.06520	0/72	N/A	1.00000	58/33370
*FANCF*	0/151	N/A	1.00000	0/233	N/A	1.00000	0/72	N/A	1.00000	21/33370
*FANCG*	0/151	N/A	1.00000	2/233	**5.89**	**0.04889**	0/72	N/A	1.00000	49/33370
*FANCI*	1/151	0.90	0.69582	2/233	1.17	0.69043	1/72	1.90	0.41266	245/33370
*FANCL*	0/151	N/A	1.00000	1/233	1.79	0.43123	0/72	N/A	1.00000	80/33370
*FANCM*	2/151	2.04	0.26091	0/233	N/A	0.41155	0/72	N/A	1.00000	218/33370
*PALB2*	0/151	N/A	1.00000	0/233	N/A	1.00000	0/72	N/A	1.00000	113/33370
*RAD51C*	2/151	7.00	0.03583	1/233	2.24	0.36410	0/72	N/A	1.00000	64/33370
*FA_combined*	9/151	0.99	0.57945	21/233	1.46	0.07000	3/72	0.64	0.80130	2002/33370
*DKC1*	0/151	N/A	1.00000	0/233	N/A	1.00000	0/72	N/A	1.00000	31/33370
*NOP10*	0/151	N/A	1.00000	0/233	N/A	1.00000	0/72	N/A	1.00000	14/33370
*POT1*	0/151	N/A	1.00000	0/233	N/A	1.00000	0/72	N/A	1.00000	59/33370
*TERT*	0/151	N/A	1.00000	1/233	2.52	0.33230	1/72	8.23	0.11760	57/33370
*TERC*	0/151	N/A	1.00000	0/233	N/A	1.00000	0/72	N/A	1.00000	0/33370
*TINF2*	0/151	N/A	1.00000	0/233	N/A	1.00000	0/72	N/A	1.00000	27/33370
*TELO_combined*	0/151	N/A	0.27713	1/233	0.38	0.52778	1/72	1.24	0.55638	374/33370

OR, Odds ratio; AA, aplastic anemia; MDS, myelodysplastic syndrome; AML, acute myeloid leukemia, Fisher's exact test used to calculate *p*-values.

When all coding frames of telomerase machinery genes (Supplementary Table 1) were analyzed, none of the 5 different variants in *TERT* previously reported to be pathogenic in hereditary AA [16] were found in our MDS and AA cohorts. However, combined, new 2 *tier-1 TERT* variants showed higher frequency in AML (*P* > 0.1; OR = 11), but without statistical significance, partially because of small sample size. Variants previously reported to be associated with AA (Ala1062Thr and His412Tyr) were either not present or not increased in their frequency in AA as compared to controls, a finding in contrast with previous reports [16–18] (Supplementary Table 3).

### Characterization of clinical phenotypes and molecular spectrum of carriers of germline variants in Fanconi Anemia and telomerase complex genes

Subsequent analysis focused on clinical features of carriers of FA and telomerase variants. The presence of heterozygous GL mutations in FA was associated with a higher frequency of somatic deletion of chr5 (3.6 OR, *P* < .0.039) and deletion of chr7 (OR 2.3, *P* = 0.08). We also observed lower rates of normal cytogenetics, higher prevalence of complex karyotype and trisomy 8, but due to small sample size none of them reached statistical significance. FA carrier status among MDS/AML patients showed no impact on survival (Supplementary Figure 2), neither association with age or clinical laboratory parameters (Table 3). We also tested the association of individual GL variants with the occurrence of specific somatic mutations, but no specific association was observed (list of somatic mutations in Supplementary Figures 3 and 4). However, when analyzed as a group, FA variant carriers were associated with the absence of *SF3B1* mutations and the presence of *PRPF8* mutations (Supplementary Figure 3). Overall, frequencies of specific types of somatic mutations (frameshift *vs.* missense/nonsense) or the total number of observed mutations were not statistically different between FA carrier and non-carrier groups. Neither family history of leukemia nor higher rates of other cancers were found.

**Table 3:** Clinical characteristics of MDS patients with respect to the presence of FA germline variants

	MDS WT FA (*N* = 188)	MDS Mutant FA (*N* = 16)	*P* value
Age (median)	65	65	1.000
Sex (Male, %)	54% (102)	56% (9)	0.757
**Cytogenetics**			
Normal	37% (69)	18% (3)	0.180
**del(5q),-5**	**7% (14)**	**25% (4)**	**0.039**
del(7q),-7	11% (19)	25% (4)	0.087
-Y	2% (3)	0% (0)	1.000
del(20q),-20	8% (15)	6% (1)	0.385
+8	4% (13)	12% (2)	0.501
Complex(≥3)	18% (35)	26% (4)	0.491
others	14% (27)	32% (5)	0.066
WBC (1,000/mm3)	3.9	3.7	*1.000*
ANC (1,000/mm3)	1.9	1.7	*1.000*
BM blast (%)	4.6	5.0	1.000
Plt (1,000/mm3)	115	124	*0.720*
OS (months)	21	18	0.070
Somatic mutations	1.4	1.6	0.828

Significant associations were put in bold.

## DISCUSSION

To date, few hereditary factors have been described for seemingly sporadic adult MDS and other BMF syndromes. We hypothesized that GL alterations may be genetic predisposition traits that in a heterozygous configuration are associated with long disease anticipation. Classical FA is an autosomal recessive disease with various physical sequelae and presumed asymptomatic heterozygous carriers. Most reports do not associate the presence of heterozygous FA variants to the increased risk of cancer or development of BMF such as AA or MDS [27–30]. Telomerase gene mutations, previously reported in AA, appear to be autosomal dominant with variable penetrance.

Our study provides the first evidence that heterozygous FA gene carriers may have delayed onset and increased risk for the development of MDS and AML, implying that the normal alleles may not be entirely protective. The observed alterations included 1 recurrent nonsense heterozygous mutation (*FANCC*), 2 recurrent frameshift, heterozygous mutations (*FANCG*) and 1 splicing variant (*FANCA*). Interestingly, FA alterations were associated with both MDS and AML, consistent with the pathophysiologic overlap between MDS and AML. The functionally detrimental impact of *tier-1* heterozygous alterations has been predicted using consistent results from an array of 6 prediction tools as well as public Fanconi Anemia databases and other resources. The molecular impact of some of the identified variants needs to be tested, since only one variant (e.g., *FANCC* p.R185X) was previously found in patients with FA (Rockefeller Fanconi mutation database [20]).

Consistent with our results, in another publication, relatives of FA carriers showed an increased rate of breaks per cell (using standard DEB and MMC testing), although the frequency of aberrant cells was lower [31] suggesting that the penetrance of the damaged allele is low, thus explaining the long latency needed to accumulate critical numbers of aberrant cells. Another interesting observation of our cohort is the enrichment of del5 and del7 alterations among FA carriers identified consistent with the high frequency of -7/del7q found among typical FA patients who develop MDS/AML.

Similar to DC associated with BMF telomeres shortening [32–34], telomerase complex genes have previously been implicated in some forms of idiopathic AA [16–18]. Previously, telomerase variant frequencies in AA were estimated at 1.5% [35] to 4.0% [16]. However, in our cohort, we identified roughly identical rates of *tier-1* alterations in MDS, AML, and AA when compared to large control populations currently available in public databases (http://exac.broadinstitute.org/; date accessed [July 2016]). For example, His412Thr (rs34094720) was previously reported to be present [16] in 1% of AA patients and 0% of controls. Yet with today's access to vast genetic databases we know that this variant is present in nearly ~2% of healthy, non-Finnish Europeans. Usage of an extended set of controls, genotyped in unbiased fashion, enabled finer disease-risk calculation and thus some of the former GWAS approaches may require careful reconsideration. While some of the variants described in AA (*TERT* His412Thr and Ala202Thr) may have shown a negative impact on telomere maintenance using *in vitro* assays, given the comparable frequencies of these variants in controls, previous studies might have captured disease-risk independent factors that could attribute to disease course/severity or play a risk factor role in the hematopoietic cell transplantation [36].

In sum, our study of the investigation of FA and telomerase gene alterations suggests that heterozygous carriers of certain FA genes may have increased risk of myeloid neoplasia but not AA and thus conversely, some of the seemingly sporadic cases of MDS may have FA-related genetic background.

## MATERIALS AND METHODS

### Patients

Tumor DNA was obtained from patient's bone marrow and/or peripheral blood. Written informed consent for sample collection was obtained according to protocols approved by the Institutional Review Board of the Cleveland Clinic and in accordance with the Declaration of Helsinki. Disease diagnoses were assigned according to the World Health Organization (WHO) classification criteria. The clinical characteristics of patients investigated in this study are presented in Table 1.

### Population frequencies for detected variants, functional annotation and variant prioritization

We used Exome Aggregation Consortium (ExAC), Cambridge, MA (URL: http://exac.broadinstitute.org) [June, 2015]. All the variants from tested genes were extracted for further functional annotation and processing. Sequencing results were annotated using ANNOVAR, http://annovar.openbioinfromatics.org. Mutations that were predicted to be damaging/probably damaging by 4/5 algorithms (PhyloP, SIFT, PolyPhen2, LRT and MutationTaster) were prioritized for further analysis (*tier1*). This functional pre-processing analysis was also carried out on control population variants that were extracted from ExAC database. Ethnically matched population and the variants extracted that we analyzed were derived from ExAC database consisting 33370 normal, White/Caucassian individuals. For the purpose of this analysis, the candidate alterations were sub-classified to prioritize known disease-prone sequence alterations/mutations (OMIM/Rockefeller Fanconi mutation database)

### Whole exome sequencing

Whole exome capture using SureSelect Human All Exon 50 Mb kit was used for targeted, exome capture according to the manufacture's protocol (SureSelect^®^, Agilent Technology, Santa Clara, CA), according to the manufacturer's protocols. The captured targets were subjected to massive sequencing using Illumina HiSeq 2000 (Illumina, INC., San Diego, CA). Generation of .bam files with its preprocessing and detection of somatic point mutations/insertions and deletions was according with GATK best practices.

### Targeted sequencing

Targeted sequencing was completed using TruSeq Enrichment library (Illumina). Two panels were created, one for somatic mutations and one for GL variants. Somatic variants were reported previously [37]. List of genes targeted can be found in Supplementary Table 1. The enriched targets were subjected to massive sequencing using Illumina Miseq sequencer, with average depth of 500×. Only variants with minimum depth at least 20 and 8 positive, high quality reads were considered for further analysis. Alterations with Variant Allelic Frequency below 35% were removed being considered somatic variants.

### Statistical analysis

For comparison between groups, with and without FA mutations, statistical analysis was performed using standard, unpaired, two sample *t*-test (for continuous, normally distributed variables) and Fisher's Exact test (for discrete data). Reported p-values here are not adjusted for multiple comparisons. Odds ratio were calculated as follows: number of patients with damaging variant multiplied by a number of normal individuals without damaging variants and divided by number of normal individuals with damaging variant multiplied by number of patients without damaging variants.

## SUPPLEMENTARY MATERIALS FIGURES AND TABLES







## Abbreviations

Chr: chromosome
RUNX1: Runt Related Transcription Factor 1
CEBPA: CCAAT/Enhancer Binding Protein Alpha
GATA2: GATA Binding Protein 2
FANC: Fanconi Anemia Complementation Group
TERT: Telomerase Reverse Transcriptase
TERC: Telomerase RNA Component
BRCA2: BRCA2: DNA Repair Associated
