# Supplementary material for: Fanconi Anemia germline variants as susceptibility factors in aplastic anemia, MDS and AML
Source: Oncotarget. 2017 Dec 16;9(2):2050–7. doi: 10.18632/oncotarget.23328 (PMC5788620; doi:10.18632/oncotarget.23328)
Supplement: Supplementary file 1 [file oncotarget-09-2050-s001.pdf]

Fanconi Anemia germline variants as susceptibility factors in aplastic anemia, MDS and AML

SUPPLEMENTARY MATERIALS

Supplementary Table 1: List of genes selected to be sequenced in the project. All coding frames, excluding 5′ and 3′ UTR were selected. See Supplementary_Table_1

Supplementary Table 2: Summary table with all detected variants listed, stratified by individual patients. Functional and location data presented in each column. The predicted function of the variants is indicated as D: damaging; N, neutral; T, tolerated; P, probably damaging; M, medium damaging; L, low damaging, A, disease automatic. See Supplementary_Table_2

Supplementary Table 3: Previously reported variants, with their respective initial frequencies, for *TERT* gene and their current reported frequencies in publicly available database (Exac database)

Gene	Variant	Exac_all_database	Previously published
TERT	Ala1062Thr	2.500%	Calado 2011 (2.6% *N* = 5), Yamaguchi 2005 (2.5% *N* = 5)
TERT	Pro530Leu	0.002%	Calado 2011 (0.4% *N* = 1)
TERT	Ala202Thr	0.060%	Yamaguchi 2005 (1% *N* = 2)
TERT	His412Tyr	1.200%	Yamaguchi 2005 (1% *N* = 2), Calado 2009 (0.5% *N* = 1)
TERT	Val1090Met	0.001%	Yamaguchi 2005 (1% *N* = 2)
TERT	Val299Met	0.010%	Calado 2009 (0.5% *N* = 1)

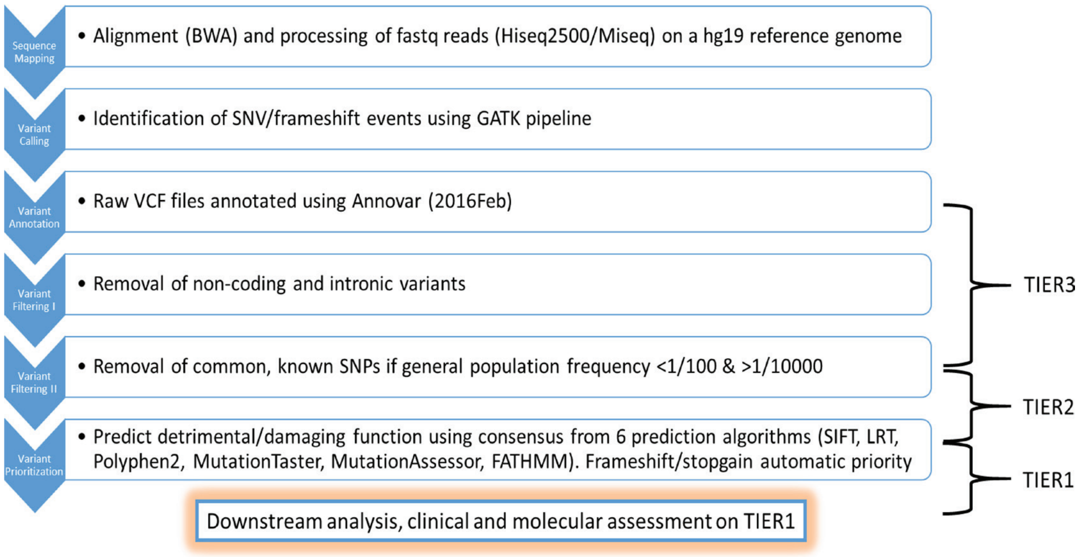

Supplementary Figure 1: Analytical pipeline used to prioritize germline, damaging variants.

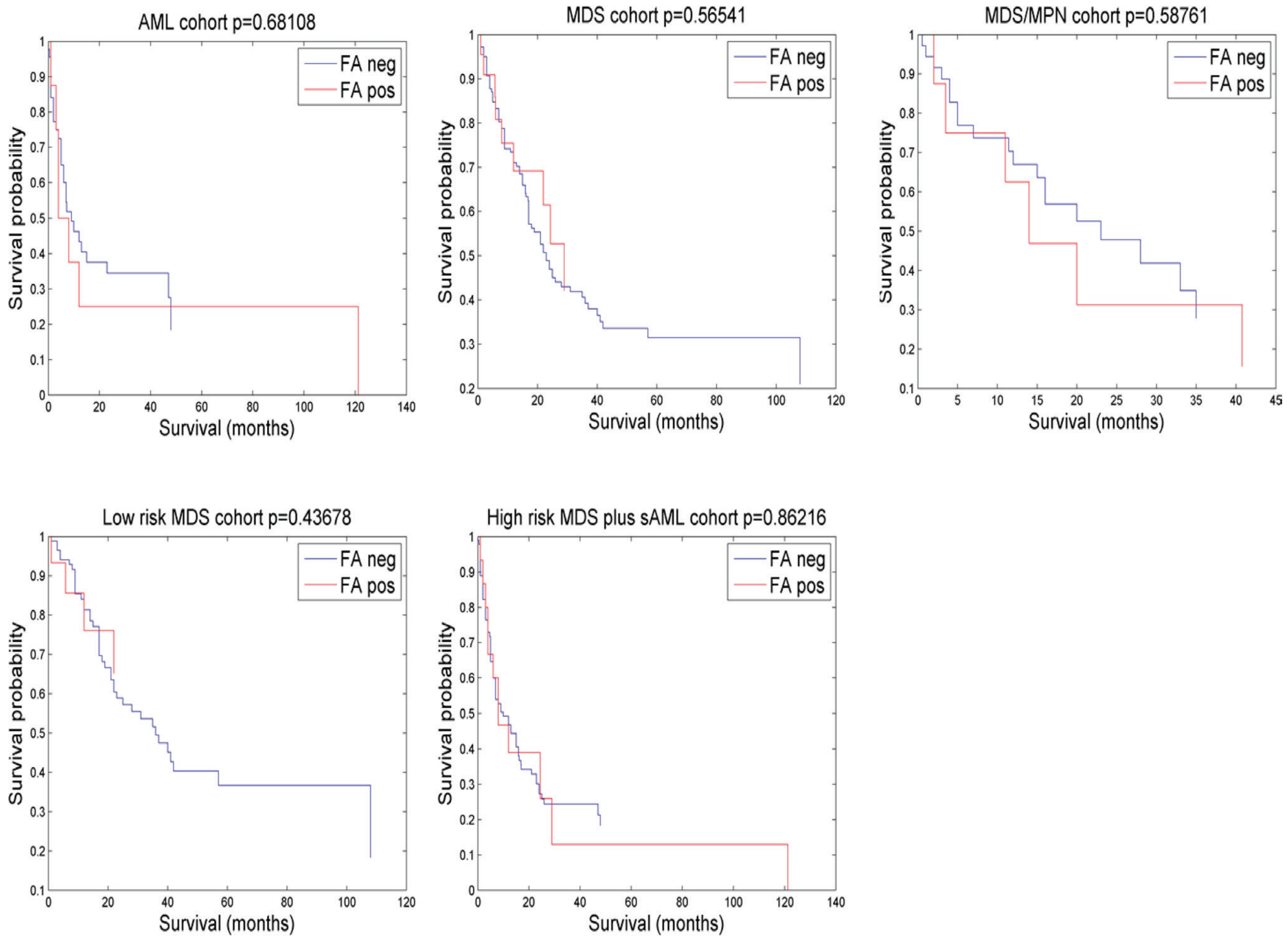

Supplementary Figure 2: Kaplan-Meier plots show the survival of patients positive for FA variants. Red line represents the survival curve for FA carriers, while blue line represents the survival curve for non-FA carriers. Each subgroup tested is indicated on top of each survival plot.

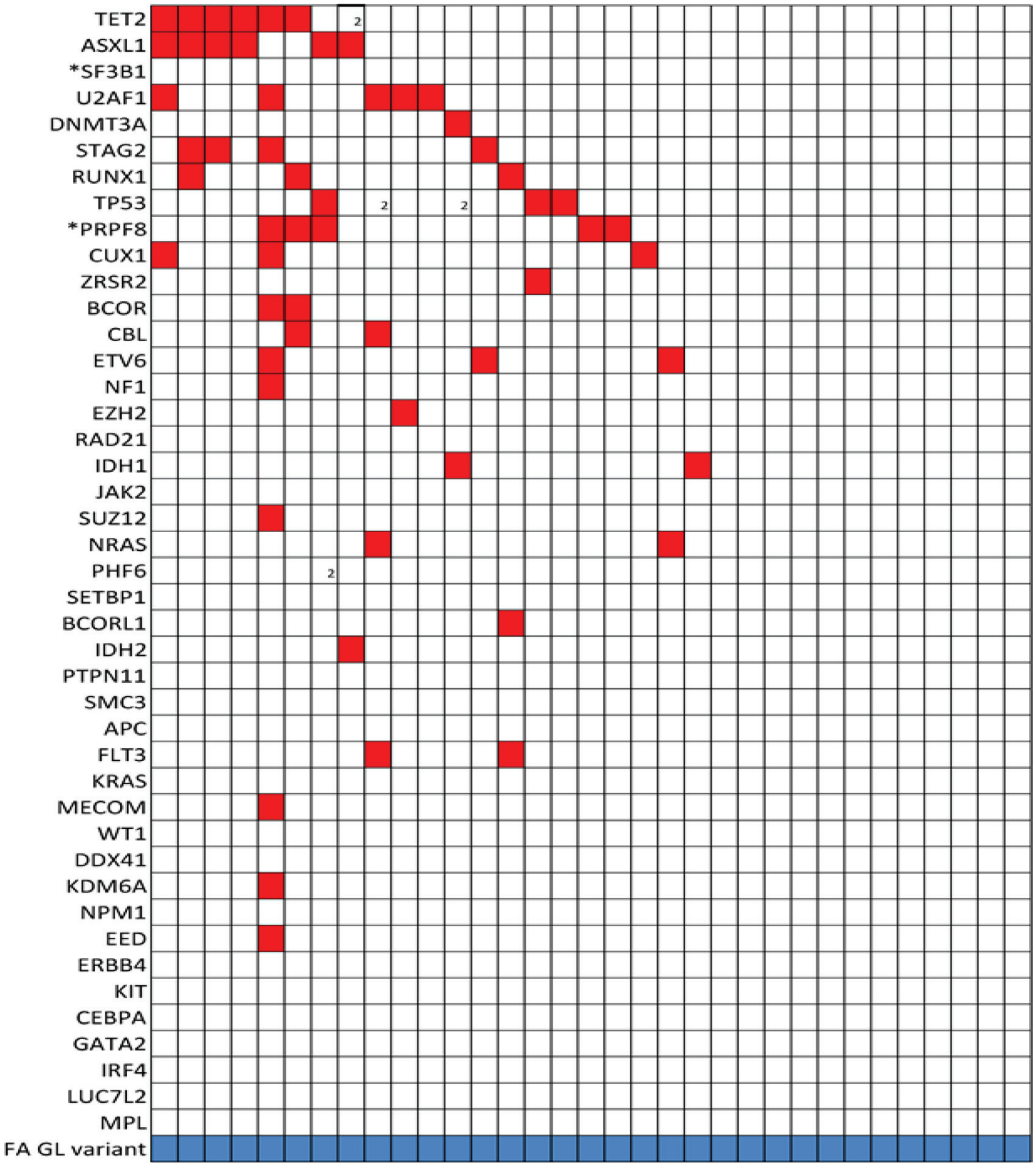

Supplementary Figure 3: Somatic landscape of patients positive for FA variants. Data for each patient is represented by columns of the matrix, rows represent each gene and its respective mutations. Red color represents patients positive for a somatic mutation within given gene. Numerical symbol ”2” represents somatic deletion of the locus.

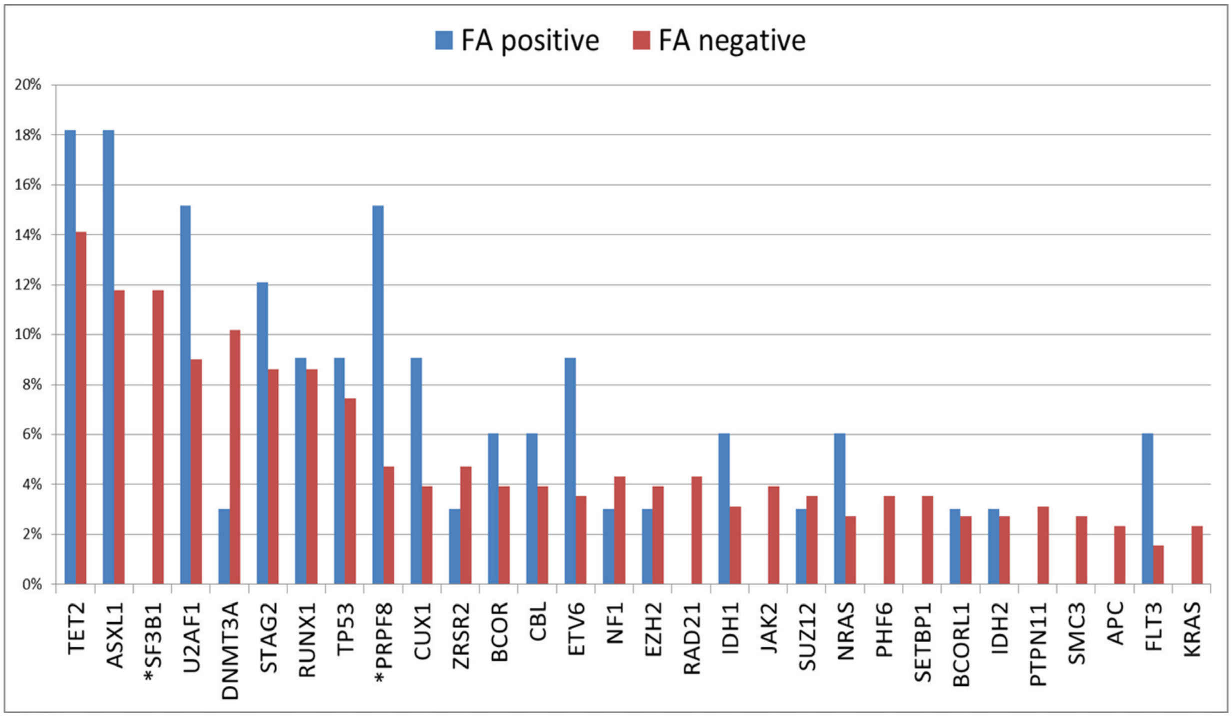

Supplementary Figure 4: Frequencies of somatic mutations between FA positive and FA negative patients. Height of the bar indicates frequency of somatic events for a given gene. Red color indicates frequency for non-FA carriers, blue represents FA carriers. Asterisk denotes significant difference between FA and non-FA (using Fisher's Exact test).
